# Are contrast enhanced mammography and digital breast tomosynthesis equally effective in diagnosing patients recalled from breast cancer screening?

**DOI:** 10.3389/fonc.2022.941312

**Published:** 2022-11-24

**Authors:** Natalia Siminiak, Anna Pasiuk-Czepczyńska, Antonina Godlewska, Piotr Wojtyś, Magdalena Olejnik, Joanna Michalak, Piotr Nowaczyk, Paweł Gajdzis, Dariusz Godlewski, Marek Ruchała, Rafał Czepczyński

**Affiliations:** ^1^ Department of Endocrinology, Metabolism and Internal Diseases, Poznan University of Medical Sciences, Poznań, Poland; ^2^ Cancer Prevention and Epidemiology Center, Poznań, Poland; ^3^ Breast Surgical Oncology Department, Greater Poland Cancer Center, Poznań, Poland

**Keywords:** breast cancer, contrast enhanced mammography, digital breast tomography (DBT), mammography, imaging modalities

## Abstract

**Purpose:**

Full-field digital mammography (FFDM) is widely used in breast cancer screening. However, to improve cancer detection rates, new diagnostic tools have been introduced. Contrast enhanced mammography (CEM) and digital breast tomosynthesis (DBT) are used in the diagnostic setting, however their accuracies need to be compared.

The aim of the study was to evaluate the diagnostic performance of CEM and DBT in women recalled from breast cancer screening program.

**Methods:**

The study included 402 consecutive patients recalled from breast cancer screening program, who were randomized into two groups, to undergo either CEM (202 patients) or DBT (200 patients). All visible lesions were evaluated and each suspicious lesion was histopathologically verified.

**Results:**

CEM detected 230 lesions; 119 were classified as benign and 111 as suspicious or malignant, whereas DBT identified 209 lesions; 105 were classified as benign and 104 as suspicious or malignant. In comparison to histopathology, CEM correctly detected cancer in 43 out of 44 cases, and DBT in all 33 cases, while FFDM identified 15 and 18 neoplastic lesions in two groups, respectively. CEM presented with 97% sensitivity, 63% specificity, 70% accuracy, 38% PPV and 99% NPV, while DBT showed 100% sensitivity, 60% specificity, 32%, PPV, 100% NPV and 66% accuracy. The CEM’s AUC was 0.97 and DBT’s 0.99. The ROC curve analysis proved a significant (p<0.000001) advantage of both CEM and DBT over FFDM, however, there was no significant difference between CEM and DBT diagnostic accuracies (p=0.23).

**Conclusions:**

In this randomized, prospective study CEM and DBT show similar diagnostic accuracy.

## Introduction

Mammography (Full Field Digital Mammography, FFDM) is the most common and widely available imaging modality for breast cancer diagnosis. FFDM is used in routine screening, in symptomatic patients, and often in monitoring primary systemic breast cancer therapy, as well as in the follow-up after treatment (1). In 2011, Food and Drug Administration (FDA) approved two new modalities: contrast enhanced mammography (CEM) and digital breast tomosynthesis (DBT) for clinical use as adjuncts to mammography (2, 3).

CEM is a promising diagnostic technique, especially in women with dense parenchymal tissue (4). By administering an intravenous contrast agent, CEM adds new physiological information to the morphological data provided by FFDM and improves the parameters of breast cancer detection (5). In a recent meta-analysis, performed on 60 studies including 11049 examinations, Cozzi et al. reported CEM sensitivity of 95% and specificity of 81% (6). The negative predictive value (NPV) of CEM reaches 100%, and the positive predictive value (PPV) varies from 93 to 97% (7, 8). In contrast, FFDM’s sensitivity remains at the level of 86-89% in low density, fatty breasts and around 62-68% in dense breasts (1).

Digital breast tomosynthesis (DBT) is a subtype of mammography, which uses the same X-ray source and creates multiple 2D images to obtain a 3D breast reconstruction (3, 9). DBT outperforms conventional mammography mostly due to the ability to evaluate overlapping breast tissue and visualize tumors, which are not visible on FFDM. Researchers estimated that the sensitivity of DBT is higher than that of FFDM, around 81.1% vs 60.4% respectively (10).

Both CEM and DBT are relatively recent/new imaging techniques and are constantly being developed. Imaging protocols, radiation dose, or image acquisition are being improved for better sensitivity, specificity, but also patient safety (11). In order to introduce new techniques to routine screening and diagnosis, it is important to assess their diagnostic value.

This paper presents novel data on CEM and DBT diagnostic efficiency. The purpose of the study was to evaluate the diagnostic performance of CEM and DBT in a cohort of women recalled from the national mammography screening program.

## Material and methods

### Patients

The study is a prospective, randomized trial, approved by the regional Medical Bioethical Committee of Greater Poland Medical Chamber. Patients involved in the study were invited to participate after being recalled from routine breast cancer screening. Patients aged from 50 to 69 years, recalled from screening, were randomized with allocation ratio 1:1 into two groups in which either CEM of both breasts or DBT of suspected breasts were performed. Patients were allocated to each group using computer-assisted randomization. The study was performed at the Cancer Prevention and Epidemiology Center in Poznań between July 2020 and September 2021. The written consent of each patient was obligatory for participation. Exclusion criteria were: signs and symptoms of breast cancer, previous breast surgery including implants, eGFR< 30 ml/min x 1.73 m2, history of allergy to iodinated contrast agents, lack of patient’s consent to participate in the trial.

### Full field digital mammography

FFDM was performed in all of the patients in a screening setting. All examinations were performed at the Center of Cancer Prevention and Epidemiology in Poznan, either in mobile (Mammomat Fusion, Siemens Healthcare, Germany) or stationary mammography units (Mammomat Inspiration Prime and Mammomat Revelation, Siemens).

All of the devices placed in mobile units were equipped with a flat panel detector with a cesium iodide scintillator, field size 230x300 mm, a 2790 × 3580 image matrix with a detector elements pitch of 83 µm.

### Contrast enhanced mammography

CEM was performed in recalled patients 2-3 weeks after the screening mammograms. All of the contrast enhanced examinations were performed by means of a stationary mammography unit (Mammomat Revelation, Siemens). It consisted of a current FFDM system using a flat panel detector (amorphous selenium (aSe)) with a field size 299×238 mm, a 2800×3518 image matrix with a detector elements pitch of 85 µm and specific software and hardware for rapid acquisition and processing of dual-energy images.

Low energy images were acquired 2 minutes after intravenous iopromide 370 (Ultravist 370, Bayer Healthcare, Berlin, Germany) administration. It was a one-shot injection using a power injector Dual Shot alpha 7 (Nemoto) of 1,5 ml/kg at a rate of 3 ml/s with a 30 ml saline bolus chaser. CEM examinations began with a mediolateral oblique (MLO) view of the breast without suspicious lesion following the breast with the suspicious lesion in order to secure more contrast uptake. The pair of low and high energy images (LE and HE) was performed on each view (MLO and cranio-caudal (CC)). Then recombined images (RC) were generated in order to visualize enhancing lesions and eliminate unenhancing background tissue. Tungsten anode material was used for all acquisitions, with a rhodium filter with kVp ranging from 25 to 32 used for low energy acquisitions similar to those in FFDM. The paired high-energy images were acquired at a 49 kVp titanium (Ti) filter in the X-ray beam to produce an X-ray spectrum above the K-edge of iodine (33.2 KeV), to increase the visibility of low concentrations of iodine (12). The entrance dose varied from 1.26 to 12.07 mGy, depending on the thickness of the breast (10 - 82 mm) and tissue composition.

All of the lesions visible in CEM were histopathologically verified, either by core needle biopsy or vacuum-assisted biopsy under ultrasound or mammography guidance.

### Digital breast tomosynthesis

DBT procedures were performed 2-3 weeks after screening mammograms, using a digital mammography Mammomat Inspiration device (Siemens). It consisted of a current FFDM system using a flat panel amorphous selenium (aSe) detector with a field size of 299 x 238 mm, a 2800 ×3518 image matrix with a detector elements pitch of 85 µm, and specific software and hardware for rapid acquisition and processing of tomographic images.

The tube swivel range was 50° (+/-25°) with 25 projections and with a 1mm distance between reconstructed slices. Two views of the breast with the suspicious lesion(s) were performed, mostly mediolateral oblique (MLO) and CC. The entrance dose varied from 2,46 to 14,92 mGy, depending on the thickness of the breast (23 - 86 mm) and tissue composition.

All of the lesions visible on DBT were biopsied and histopathologically proven.

### Image evaluation

Vue PACS review workstation (Carestream) was used for image analysis. Two radiologists with 8 and 15 years of experience in breast imaging evaluated the recalled patients either with DBT or CEM. In practice, each CEM and DBT examination was evaluated independently by each radiologist and the results of their assessment were recorded. In case of discordance, the examination was reviewed by both readers and the consensus was recorded as the final result.

The two radiologists were corcordant in 179 cases

All the mammograms of recalled patients were evaluated according to the ACR Breast Imaging Reporting and Data System^®^ (ACR - BIRADS (13, 14)). All the recalled patients’ baseline mammograms were classified as BIRADS 0 - demanding further evaluation to be classified into adequate BIRADS category (1, 2, 3, 4 or 5), BIRADS 4 - suspicious, BIRADS 5 - malignant.

Subsequently, lesions of patients from CEM and DBT groups were reported in concordance with the CEM supplement to ACR BIRADS^®^ Mammography 2013 (14) and categorized into the following BIRADS groups: BIRADS 1 - negative, BIRADS 2 - benign, BIRADS 3 - probably benign, BIRADS 4 - suspicious, BIRADS 5 - highly suggestive of malignancy.

Patients with BIRADS 1 are being followed up with CEM or DBT respectively, performed after 12 and 24 months. All the lesions classified above BIRADS 1 were biopsied. BIRADS classification outcome was compared to histopathology results. Finally, on the basis of radio-pathological concordance, lesions were classified as:

1) true positive (BIRADS ≧̸ 4 and biopsy-proven cancer),
2) false positive (BIRADS ≧̸ 4 and biopsy-proven benign lesion),
3) false negative (BIRADS ≤3 and biopsy-proven cancer),
4) true negative (BIRADS ≤3 and biopsy-proven benign lesion).

All in all, the results of each patient group were compared (**Tables 1**, **2**).

**Table 1:** Distribution of lesions visible in CEM.

Lesions detected by CEM	
Histopathological findings	Number of lesions detected
**Benign Lesions**	61
fibrocystic changes	19
fibroadenoma	10
intraductal papilloma	6
fragments of atrophic breast tissue	4
fragments of breast tissue	4
sclerosing adenosis	4
intraductal papilloma with UDH	3
intramammary lymph node	3
fibrocystic changes with UDH	3
inflammation due to hidradenitis suppurativa (acne inversa)	2
fibroadenoma with usual ductal hyperplasia (UDH)	1
fibrocystic changes with sclerosing adenosis	1
columnar cell changes	1
**Suspicious (B3) Lesions**	6
atypical lobular hyperplasia	3
atypical ductal hyperplasia (ADH)	2
breast papilloma with ADH	1
**Cancers**	44
invasive carcinoma of no special type (NST)	30
invasive lobular carcinoma	7
invasive carcinoma of no special type (NST) with ductal carcinoma *in situ* (DCIS)	3
invasive carcinoma of no special type (NST), partially mucinous, with DCIS	1
invasive carcinoma of no special type (NST), partially micropapillary	1
invasive lobular carcinoma with lobular carcinoma in situ	1
invasive lobular carcinoma with invasive carcinoma NST and lobular carcinoma *in situ* (LCIS)	1

**Table 2:** Distribution of lesions visible in DBT.

Lesions detected by DBT	
Histopathological findings	Number of lesions detected
**Benign Lesions**	67
fibrocystic changes	32
fibroadenoma	8
usual ductal hyperplasia (UDH)	5
fibrocystic changes with UDH	4
fibrocystic changes with sclerosing adenosis and microcalcifications	3
intramammary lymph node	3
sclerosing adenosis + simple adenosis	3
fibroadenoma with microcalcifications	2
fibroadenomatous like changes	2
fibrocystic changes with UDH and apocrine hyperplasia	1
hamartoma	1
fragments of breast tissue	1
fragments of breast tissue with signs of chronic inflammation	1
nodular adenosis	1
**Suspicious (B3) Lesions**	4
atypical ductal hyperplasia (ADH)	3
breast papilloma with ADH	1
**Cancers**	33
invasive carcinoma of no special type (NST)	18
invasive carcinoma of no special type (NST) with ductal carcinoma *in situ* (DCIS)	5
ductal carcinoma *in situ* (DCIS)	4
invasive lobular carcinoma	4
mucinous carcinoma	1
invasive carcinoma of no special type (NST), partially micropapillary	1

### Statistical analysis

The calculations, including the sample size, were made using Statistica 13 by TIBCO and PQStat by PQStat Software. The level of significance was α = 0.05. The result was considered statistically significant when p<α. The normality of the distribution of variables was tested with the Shapiro-Wilk test. In order to compare the variables between the two groups, the Mann-Whitney test was calculated because of non-compliance with the normal distribution. The correlation between categorical variables was calculated using the chi² test of independence or the Fisher-Freeman-Halton test. The compliance of the methods of assessing the occurrence of neoplasms was tested by calculating Cohen’s kappa coefficient of concordance and determining their significance using the Z test. The Fleiss kappa coefficient of concordance was calculated to test the consistency of all 3 methods (mammography, CEM/DBT, and biopsy) simultaneously. Additionally, the sensitivity, specificity, PPV, and NPV were determined with 95% confidence intervals. ROC analysis was performed to calculate the optimal cut-off point for BIRADS. The area under the curves (AUC) with 95% confidence intervals was determined using the non-parametric DeLong method. The optimal cut-off point was established using the Youden Index. Sensitivity and specificity were determined for the selected cut-off. The determined areas under the curve were compared with each other using the Z statistics.

## Results

402 consecutive patients recalled from the national breast cancer screening program were included in the study. The sample size was sufficient for analysis according to the sample size power calculation (minimal sample size 151 subjects). One half of the patients (200) underwent FFDM in the screening setting followed by the CEM examination, whereas 202 patients underwent FFDM followed by the DBT examination. The two radiologists, who evaluated the images, were concordant with their diagnosis in 179 CEM cases (89.5%) and the consensus had to be achieved in the remaining 21 discordant results (10.5%). In case of the DBT, the two readers were concordant in 194 patients (96.0%) and discordant in 8 cases (4.0%).

### CEM group

**Figure 1:**
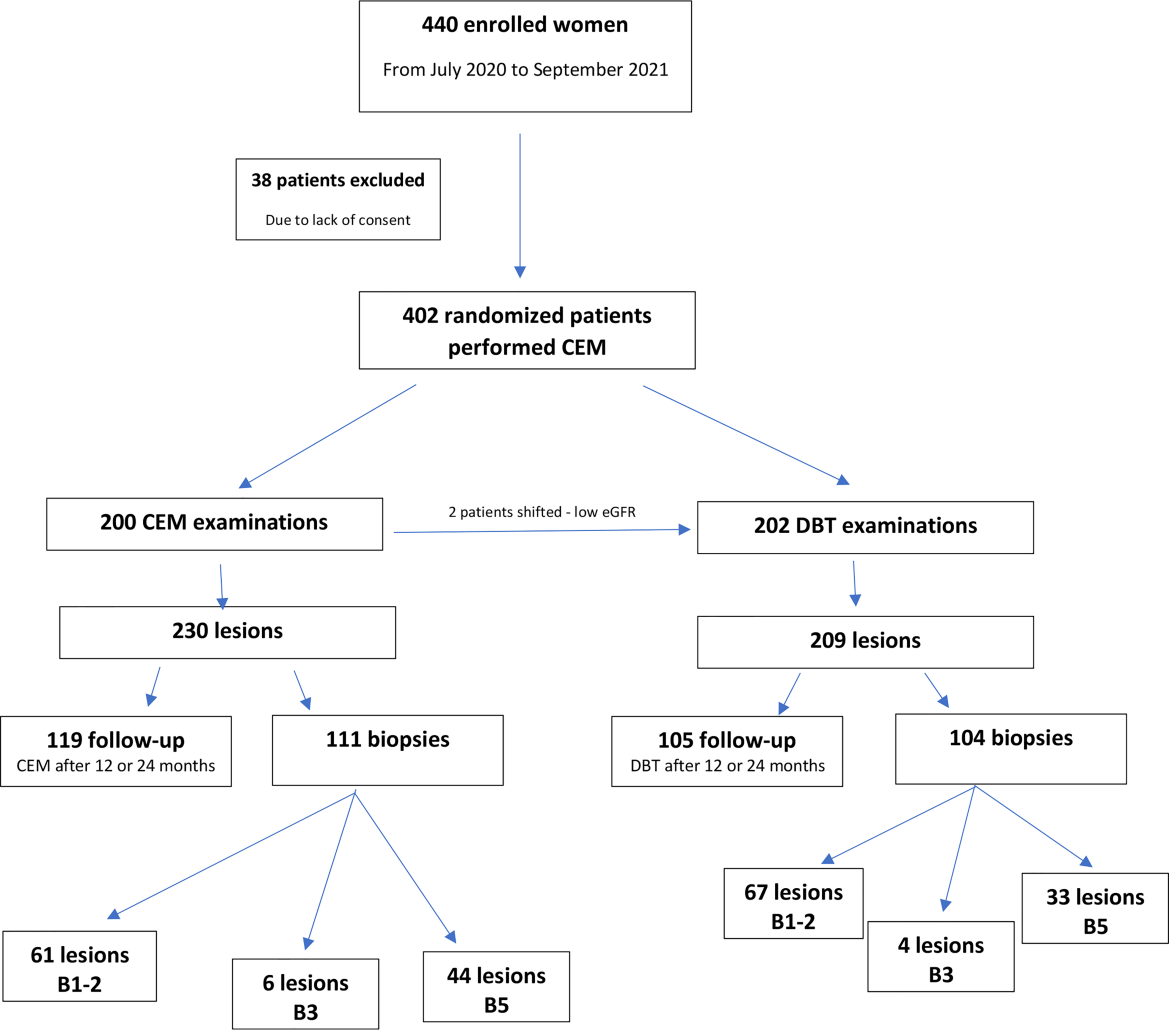
Study flowchart presenting distribution of patients and lesions due to the histopathology results estimated by core needle biopsy or vacuum-assisted biopsy under ultrasound or mammography guidance (15).

As detailed in the study flowchart (**Figure 1**), CEM indicated 230 lesions; 119 of them (52%) were described as benign (BIRADS 1 or 2) and 111 (48%) as suspicious or malignant (BIRADS ≥ 4). Histopathology examination confirmed cancer in 44 lesions. CEM was true positive in 43 cases, true negative in 118 cases, false positive in 68 cases, and there was 1 false-negative case. 

FFDM, performed in CEM subgroup patients, indicated 205 lesions, where 171 were classified as benign (BIRADS = 1 or 2) and 34 were described as suspicious or malignant (BIRADS ≥ 4). FFDM was true positive in 15 cases and true negative in 148 cases, however, it was false negative in 23 cases and false positive in 19 patients. 

CEM presented sensitivity of 97%, specificity 63%, accuracy of 70%, PPV of 38%, NPV of 99%, whereas FFDM in this subgroup showed sensitivity of 40%, specificity 87%, PPV 44%, NPV 87% and accuracy 80% (**Table 3**).

**Table 3:** Sensitivity, specificity, accuracy, PPV and NPV levels according to BI-RADS assessment. 95% confidence intervals are presented in brackets.

	FFDM in CEM group	CEM	FFDM in DBT group	DBT
SENSITIVITY	40% [24%, 57%]	97% [88%, 99%]	55% [36%, 72%]	100% [89%,100%]
SPECIFICITY	87% [83%, 93%]	63% [56%, 70%]	91% [85%, 95%]	60% [52%,67%]
ACCURACY	80% [73%, 85%]	70% [64%, 76%]	85% [79%, 90%]	66% [59%, 72%]
PPV	44% [27%, 62%]	39% [30%, 49%]	53% [35%, 70%]	32% [23%,42%]
NPV	87% [81%, 91%]	99% [95%, 99%]	91% [86%, 95%]	100% [97%, 100%]

In summary, CEM indicated 43 cancer lesions, whereas FFDM identified cancer in 15 cases.

The Kappa analysis confirmed a fair concordance level (Kappa = 0.29) between FFDM and biopsy, as well as between CEM and biopsy (Kappa= 0.39).

**Figure 2:**
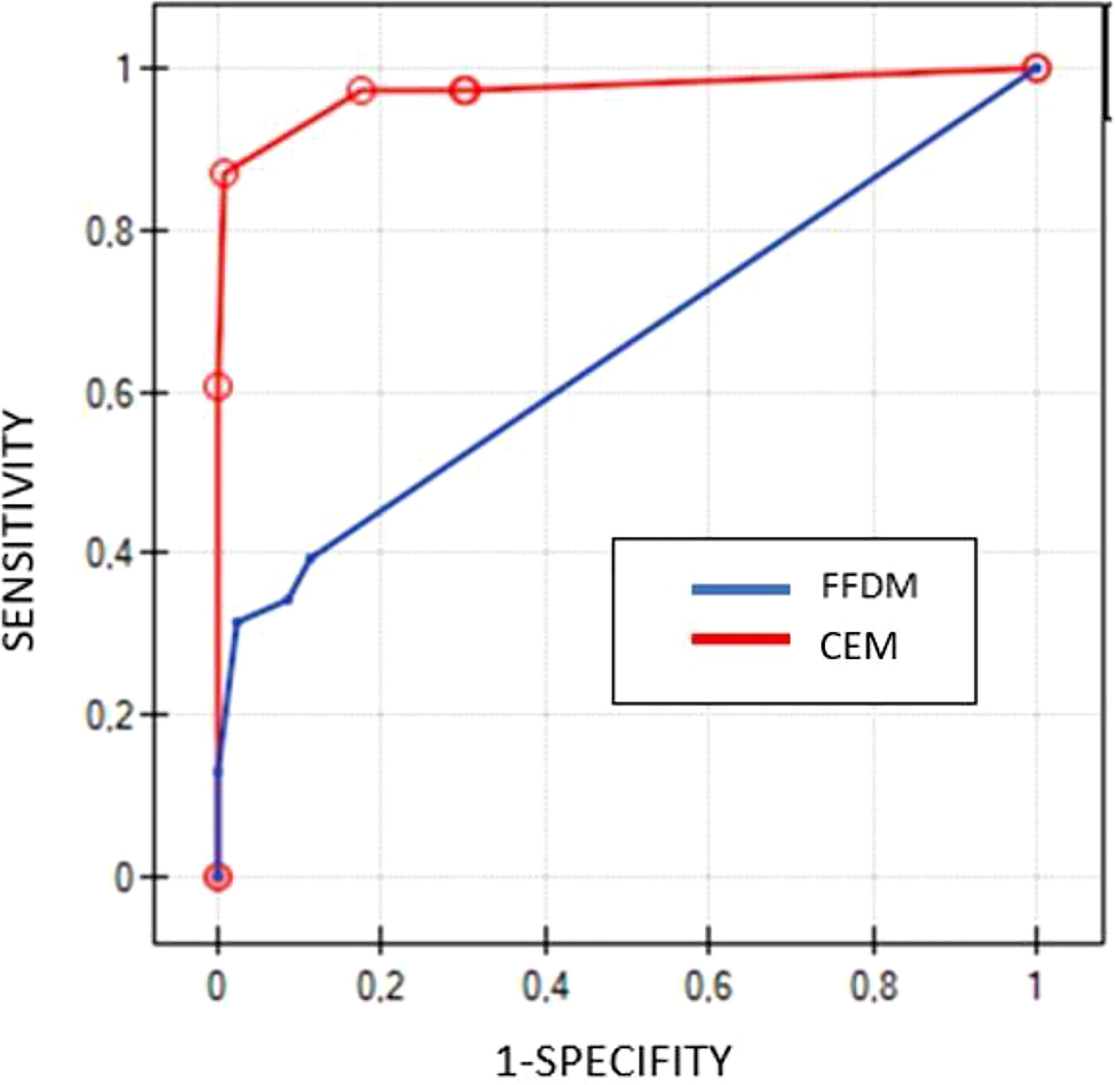
Comparison of ROC curves for CEM (red line) and FFDM (blue line) based on BIRADS scores.

The ROC curves, based on BI-RADS classification, showed significant differences between CEM and FFDM examinations (p<0.000001). CEM presented with AUC 0,97, while FFDM with AUC 0.65 (**Figure 2**).

### DBT subgroup

DBT indicated 209 lesions, where 105 cases were described as benign (BIRADS = 1 or 2) and 104 lesions as suspicious or malignant (BIRADS ≥4). Histopathology confirmed cancer in 33 tumors. DBT was true positive in 33 cases, true negative in 105 cases, and false-positive in 71 cases. DBT presented sensitivity of 100%, specificity of 60%, PPV 32%, NPV 100% and accuracy 66% (**Table 3**).

FFDM, performed prior to DBT, indicated 207 lesions, where 173 cases were described as benign (BIRADS = 1), and 34 cases as suspicious or malignant (BIRADS ≥4). FFDM was true positive in 18 cases and true negative in 157 cases, however, it was false negative in 18 cases and false positive in 16 cases. FFDM showed sensitivity of 55%, specificity of 91%, PPV 53%, NPV 91% and accuracy 85%.

In summary, DBT indicated 33 lesions as malignant and all of them were confirmed as cancers in the biopsy, whereas FFDM indicated cancer in 18 of these cases.

The Kappa analysis indicated a fair concordance level (Kappa = 0.32) between DBT and biopsy. However, a moderate concordance level (Kappa = 0.45) was established between FFDM and biopsy.

**Figure 3:**
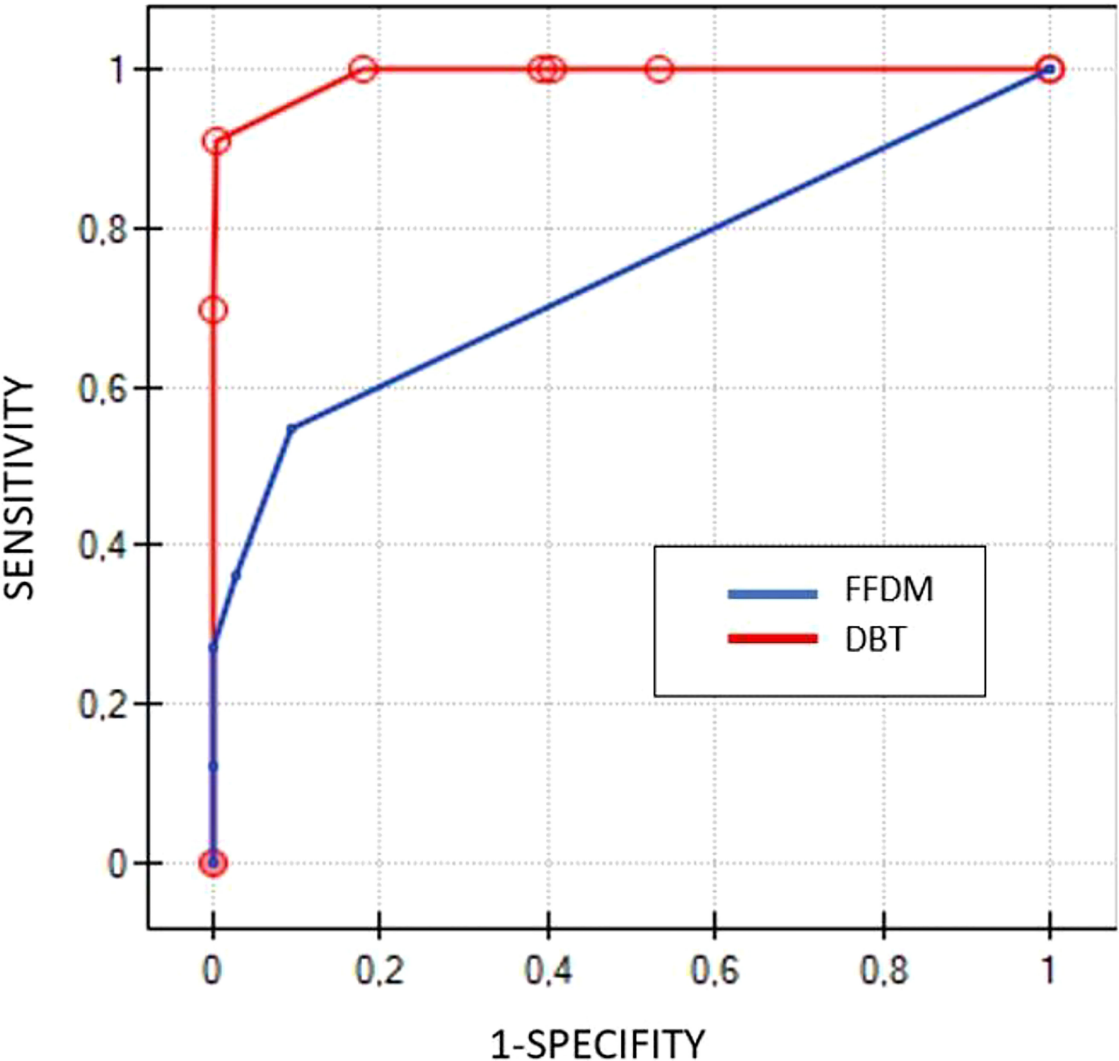
Comparison of ROC curves for DBT (red line) and FFDM (blue line) based on BIRADS scores.

The ROC curves, based on BI-RADS classification, showed significant differences between DBT and mammography examinations (p<0.000001). DBT presented with an AUC of 0.99, while mammography with AUC of 0.74 (**Figure 3**).

### The comparison between CEM and DBT groups

The groups did not present any differences in the age of patients (average 59 years) as well as in the size of lesions (average size 12.3 mm in the DBT group and 11.8 mm in the CEM group).

The cancer detection rate was similar in both modalities (p=0.8) and the percentage of diagnosed cancer cases (histopathological confirmation) in DBT and CEM were similar (p=0.35).

**Figure 4:**
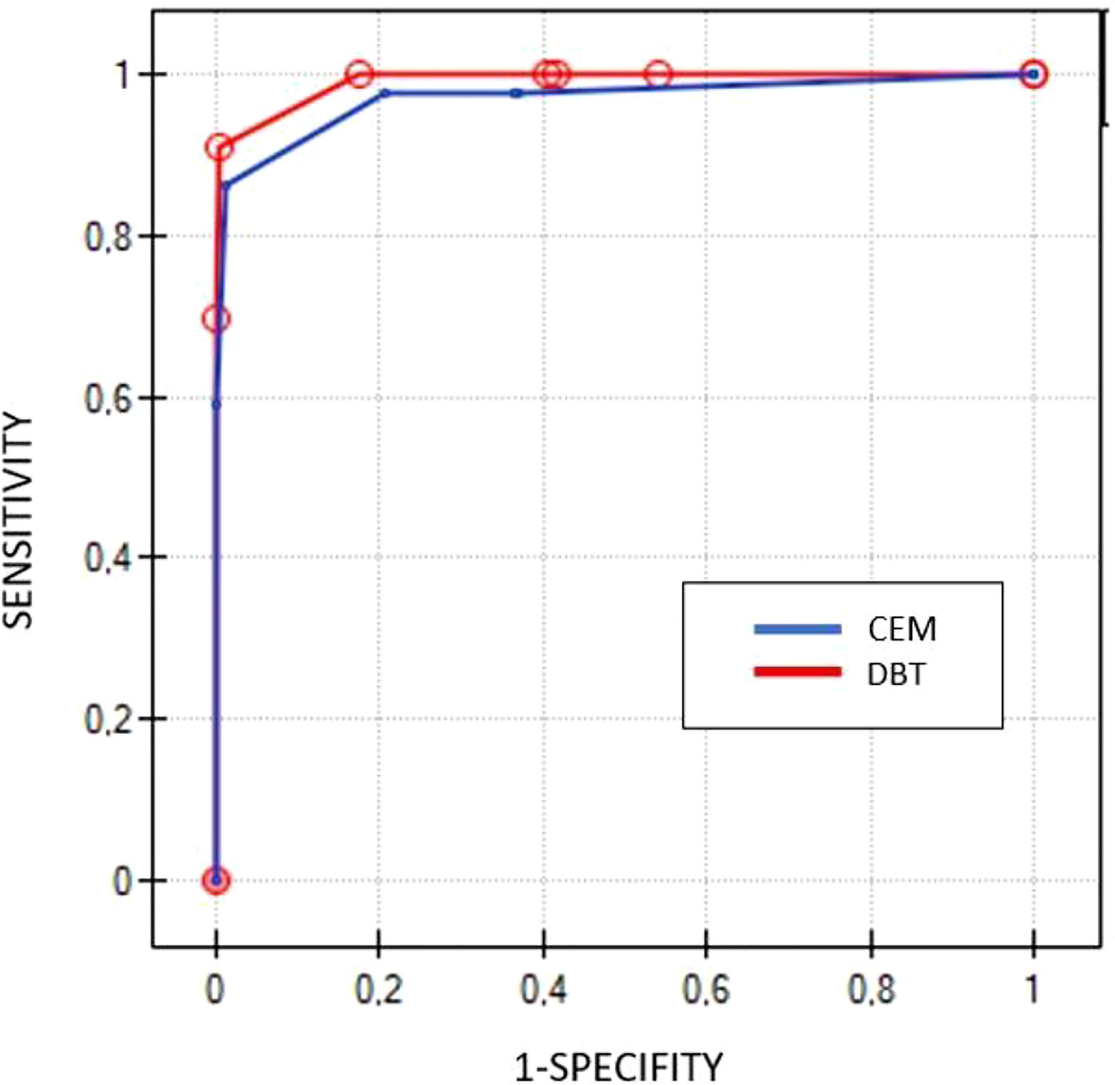
Comparison of ROC curves for DBT (red line) and CEM (blue line) based on BIRADS scores.

The ROC curves based on BI-RADS classifications for DBT and CEM are located similarly, demonstrating their similar diagnostic abilities. There were no significant differences between ROC curve areas (p=0.23) (**Figure 4**).

## Discussion

Breast cancer is the most often diagnosed malignancy and the first cause of cancer death among women. The International Agency for Research on Cancer (IARC) reported 2.26 million cases of breast cancer in 2020 worldwide (16). Early detection continues to be the key to a better prognosis and higher survival rate. FFDM remains the gold standard in breast cancer screening, however novel diagnostic tools, which may improve its accuracy (CEM, DBT, magnetic resonance), have been introduced. To the best of our knowledge, this is the first randomized, prospective study comparing CEM and DBT performance in patients recalled from breast cancer screening.

Studies have reported that both CEM and DBT have shown high sensitivity, especially with dense breasts, as compared to FFDM (17). In our study, the distribution of breast density patterns was similar in both groups. The analysis of our study cohort divided into four density patterns (A, B, C, and D according to ACR) will be performed in the further stage of our project.

The analysis confirmed high sensitivity (97%) and specificity (63%) of CEM, similar to those previously reported in the literature (2, 4, 5, 7, 8). CEM indicated 43 of 44 histopathologically verified cancer cases in our group of patients. FFDM presented with a sensitivity of 40% and specificity of around 87%, indicating 15 of 44 cancer lesions. ROC curves presented significant differences (p<0.000001) in diagnostic performance between CEM and FFDM (**Figure 2**). Despite the low concordance level of CEM and biopsy, it was slightly higher (0.39), as compared to FFDM and biopsy (0.29). The advantage of CEM over FFDM is obtained by the ability to visualize lesions obscured by dense glandular tissue. Moreover, the contrast enhancement allows to establish more accurately the size and number of lesions (1).

DBT allows viewing breast anatomy in multiple sections, which increases the diagnostic accuracy, as compared to FFDM, even if additional projections are employed (3, 9).

As predicted, the results of the DBT group also indicated an advantage of this technique over FFDM, with 100% sensitivity and specificity of 60%. DBT indicated all 33 cancers, confirmed in biopsy and there were no false-negative cases, whereas FFDM indicated 18 of them. The ROC curves, similarly to the CEM group, presented significant differences between DBT and FFDM (p<0.000001) (**Figure 3**). Surprisingly, unlike in the CEM group, the kappa test indicated moderate concordance between FFDM and biopsy (0.45), but low concordance between DBT and biopsy (0.32).

**Figure 5:**
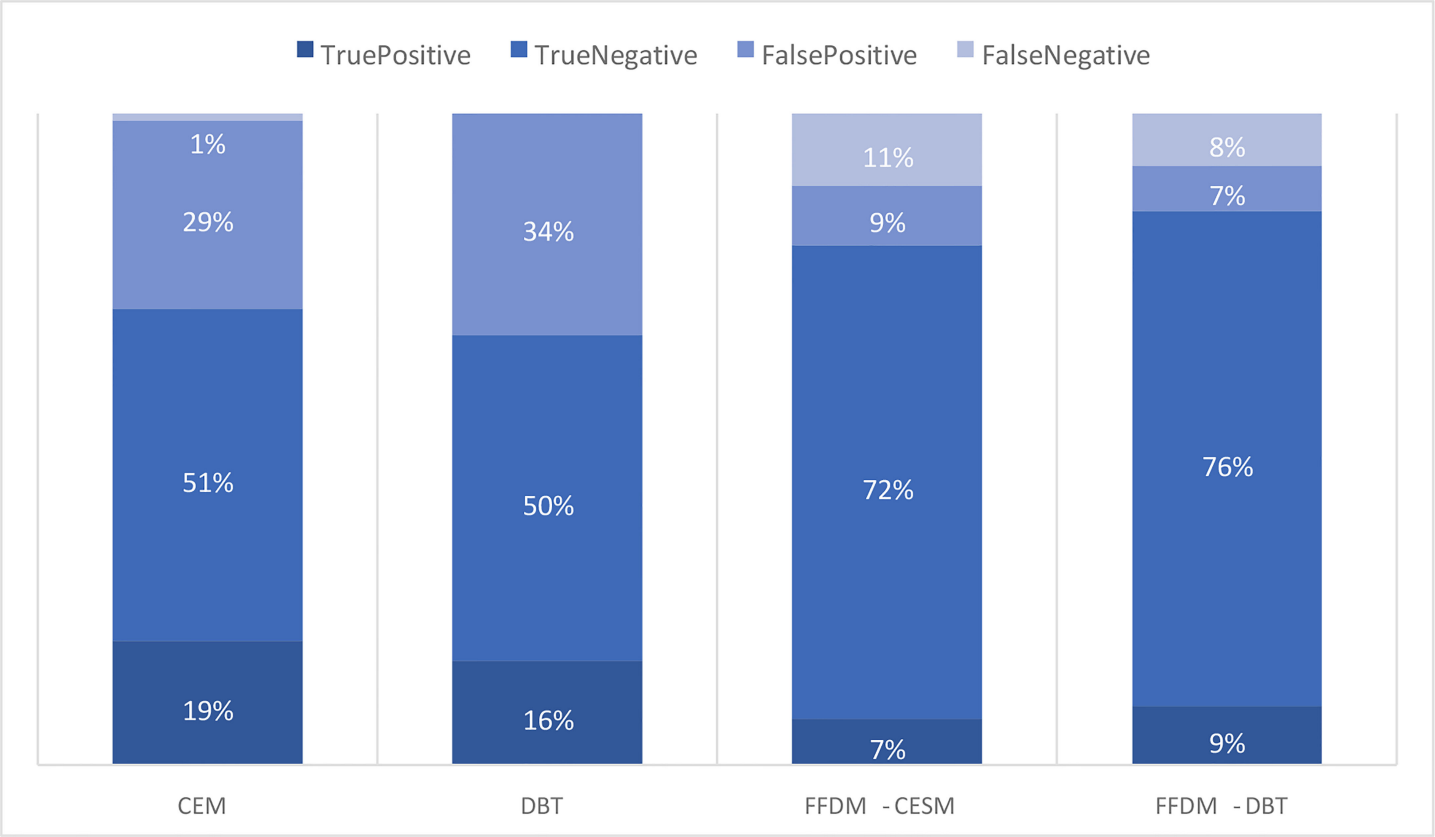
Comparison of the occurrence of true-positive, true-negative, false-positive and falsenegative results in CEM, DBT and FFDM subgroups.

Despite the 100% accuracy of DBT and 97% accuracy of CEM, the kappa test indicated a low concordance level of both modalities with biopsy. Potential explanations include a high rate of false-positive cases in DBT (34%) and in CEM (30%). FFDM in the DBT group, despite a worse cancer detection rate, showed false results in 14% of cases, including 7% of false-negative and 7% of false-positive, which caused moderate concordance with biopsy (**Figure 5**).

**Figure 6:**
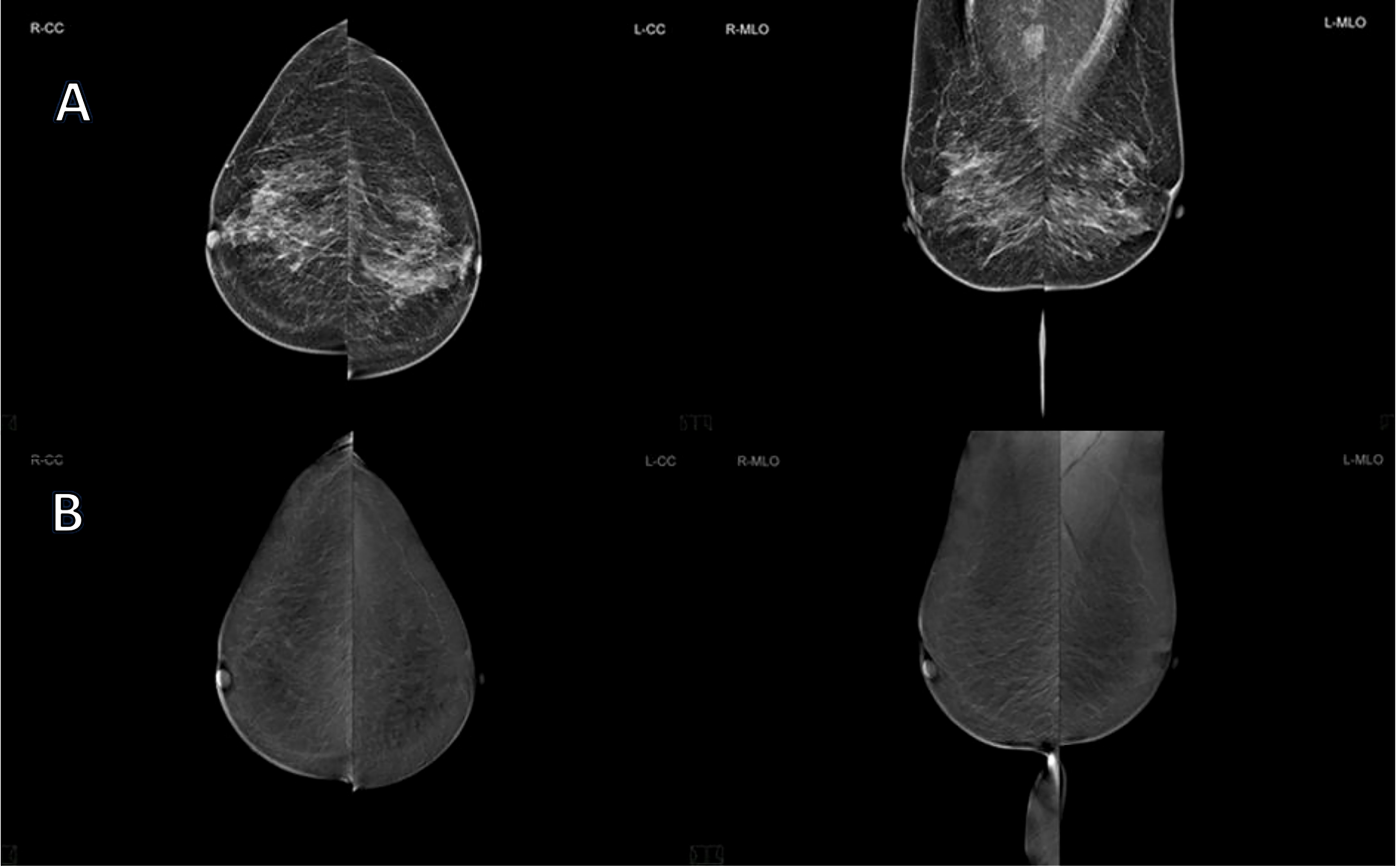
65 years old patient, with ACR type B breast density, recalled from screening due to the suspicious mass visible on RMLO in FFDM, classified as BIRADS 0 **(A)** and CEM **(B)** revealed no pathological enhancement. However, US was performed and it detected axillary lymph nodes with a typical metastatic appearance. One of them was biopsied and the pathology report revealed lymph node metastases of invasive lobular carcinoma. Breast MRI and PET-CT examinations were performed as well and they showed no signs of the primary tumor. The patient underwent neoadjuvant hormonal therapy and right mastectomy with axillary lymph node dissection. The final pathology report revealed multicentric invasive lobular carcinoma (grade 2) of the right breast with metastases to 23 out of 28 axillary lymph nodes.

It is interesting to note that the one lesion, which was indicated as a false-negative in CEM was invisible also in MR or PET/CT examinations. The patient was recalled due to enlarged axillary lymph nodes. Histopathological examination confirmed multicentric lobular cancer (EG: 75%, PG: 10%, HER2 negative, Ki67: 10%, NHG2) that might not be visible on imaging modalities such as CEM or MRI, due to its slow growth and low metabolic activity. (**Figure 6**) (5). It is not clear if the lesion would be visible in DBT. The patient had a multicentric invasive lobular cancer (ILC) which is characterized by slow growth and low metabolic activity. This is why the lesion was not detected by other modalities. DBT is the best imaging tool for the assessment of architectural distortions. Theoretically, large, slow growing ILC could manifest as a non-specific architectural distortion in DBT.

We found no significant differences between CEM and DBT (**Figure 4**). The groups were standardized, the patient’s ages and sizes of lesions were similar. What is more, there were no significant differences in cancer detection rates. These results lead to the conclusion that CEM and DBT are equivalent and may be used alternatively in patients recalled from screening. One might expect that CEM would significantly outperform DBT, due to the administration of a contrast agent and subsequent independence of breast density (4, 5, 7, 8). Moreover, CEM provides data on tumor vasculature including neoangiogenesis (18, 19). In spite of these advantages, CEM cancer detection rate in our cohort did not outperform DBT. The probable explanation for that might be in the reduction of tissue overlap due to the tomographic technique of image acquisition (20). Furthermore, the radiation dose between CEM and DBT examinations in our study was similar.

On the contrary, authors of a recent paper who evaluated 220 women examined with CEM after being recalled from screening, found that CEM-based approach outperforms standard assessment (i.e. DBT or additional mammographic views) (21). However, the cited study was based on one-arm protocol and the age range of qualified patients was different from ours, so the results are not exactly comparable. The age of 50-69 years is concordant with the European Commission Initiative on Breast Cancer guidelines of breast cancer screening. Also Girometti et al. presented a comparison of CEM and DBT+FFDM in 78 preoperative patients with histopathologically confirmed breast cancer and proved the superiority of CEM over DBT. However, it was a retrospective study comparing performance of 4 blinded readers, whereas our study is a prospective trial with a direct comparison of those two imaging tools (22). Moreover, Zuley et al. analyzed 60 lesions in 54 patients retrospectively and found that CEM reduced false-positive rate to 39% in comparison to FFDM/DBT (47%) and ultrasonography (61%) (23).

Due to the contrast injection, CEM examination is associated with a higher risk of complications, such as kidney failure or an allergic reaction. Approximately 2 in 10000 patients will develop anaphylaxis (0,15%) and 0,02-0,04% will develop severe anaphylaxis reaction. Contrast induced nephropathy develops in 1-2% patients. To prevent Post Contrast Acute Kidney Injury (PC-KAI) it is important to check eGFR level within three months before examinations and avoid repeated iodinated contrast examinations. The guidelines indicate that if the patient is under 70 years old, with no history of renal disease, proteinuria, hypertension, hyperuricemia or diabetes, then there is no need to check the eGFR level. During our study to prevent PC-AKI we evaluated creatinine and eGFR level in every patient (11, 24). On the other hand, DBT is more cost-effective and less stressful for patients (no contrast administration). Available literature focuses on the comparison of CEM or DBT with FFDM independently. To the best of our knowledge, this is the first study comparing both modalities.

Our study has certain limitations. The major limitation is the low number of participating readers. There is a limited number of experienced breast radiologists in general, also in our center. This is a single-center study and therefore, despite the extensive experience of our radiologists participating in the evaluation of images, the results should be verified in a multicenter setting. Secondly, both techniques being compared, i.e. CEM and DBT, were used in parallel groups of patients. The reason for that trial design was related to the ethical considerations of avoiding additional risk (excessive radiation). Due to the risk of contrast-induced nephropathy, two patients with abnormal eGFR were excluded from the CEM group and DBT was performed instead.

Most of the studies on CEM in the diagnosis of breast cancer were performed using mammographs produced by GE Healthcare (8, 21). There is little data in literature on the performance of mammographs manufactured by Siemens that were used in our study. It may be attributed to the relatively recent appearance of Siemens CEM technology on the market. Another technological difference is the contrast agent used for CEM. Iopromide 370 (Ultravist 370) was used in our study, whereas other authors used Iohexol 300, Iohexol 350 or Iomeprol 400. All of these agents have slightly different concentrations of iodine (11).

In our opinion, larger multicenter trials should be taken into account to confirm our results in a larger, more heterogeneous population of patients recalled from screening. Such trials should be organized possibly on an international level in order to establish the role of CEM and DBT in the management of this particular patient population independent of the influences of some local factors.

## Conclusions

To our knowledge, this is the first report of a randomized comparison of CEM and DBT. Both studied modalities demonstrated high diagnostic performance, and none of them was found to be superior. Therefore, the choice of method should be based on availability, patient’s safety and economic factors.

## Data availability statement

The original contributions presented in the study are included in the article/supplementary material. Further inquiries can be directed to the corresponding author.

## Ethics statement

The studies involving human participants were reviewed and approved by Regional Medical Bioethical Committee of Greater Poland Medical Chamber. The patients/participants provided their written informed consent to participate in this study.

## Author contributions

NS: Data analysis and interpretation. Writing the manuscript. AP-C: Data analysis and interpretation. Writing the manuscript. AG: Data collection and data analysis. PW: Data collection and statistical analysis. MO: Data collection. JM: Data collection. PN: Patients Management. PG: Patients Management. DG: Drafting the article. MR: Final approval. RC: Analysis and interpretation of results, drafting the article, final approval. All authors contributed to the article and approved the submitted version.

## Conflict of interest

The authors declare that the research was conducted in the absence of any commercial or financial relationships that could be construed as a potential conflict of interest.

## Publisher’s note

All claims expressed in this article are solely those of the authors and do not necessarily represent those of their affiliated organizations, or those of the publisher, the editors and the reviewers. Any product that may be evaluated in this article, or claim that may be made by its manufacturer, is not guaranteed or endorsed by the publisher.

## References

[1] Sardanelli F Aase HS Álvarez M Azavedo E Baarslag HJ Balleyguier. Position paper on screening for breast cancer by the European society of breast imaging (EUSOBI) and 30 national breast radiology bodies from Austria, Belgium, Bosnia and Herzegovina, Bulgaria, Croatia, Czech republic, Denmark, Estonia, Finland, France, Germany, Greece, Hungary, Iceland, Ireland, Italy, Israel, Lithuania, Moldova, the Netherlands, Norway, Poland, Portugal, Romania, Serbia, Slovakia, Spain, Sweden, Switzerland and Turkey. Eur Radiol (2017) 27(7):2737–43. doi: 10.1007/s00330-016-4612-z PMC5486792 27807699

[2] FDA. 510(k) clearance for GE contrast enhanced spectral mammography (CESM). Available at: https://www.accessdata.fda.gov/cdrhdocs/pdf10/K103485.pdf.

[3] Dhamija E Gulati M Deo S Gogia A Hari S. Digital breast tomosynthesis: an overview. Indian J Surg Oncol (2021) 12(2):315–29. doi: 10.1007/s13193-021-01310-y PMC8272763 34295076

[4] Sogani J Mango VL Keating D Sung JS Jochelson MS. Contrast-enhanced mammography: Past, present, and future. Clin Imaging (2021) 69:269–79. doi: 10.1016/j.clinimag.2020.09.003 PMC8494428 33032103

[5] James JJ Tennant SL. Contrast-enhanced spectral mammography (CESM). Clin Radiol (2018) 73(8):715–23. doi: 10.1016/j.crad.2018.05.005 29937340

[6] Cozzi A Magni V Zanardo M Schiaffino S Sardanelli F. Contrast-enhanced mammography: A systematic review and meta-analysis of diagnostic performance. Radiology (2022) 302(3):568–81. doi: 10.1148/radiol.211412 34904875

[7] Zhu X Huang JM Zhang K Xia LJ Feng L Yang P. Diagnostic value of contrast-enhanced spectral mammography for screening breast cancer: systematic review and meta-analysis. Clin Breast Cancer (2018) 18:e985–95. doi: 10.1016/j.clbc.2018.06.003 29983379

[8] Łuczyńska E Heinze-Paluchowska S Hendrick E Dyczek S Ryś J Herman K. Comparison between breast MRI and contrast-enhanced spectral mammography. Med Sci monitor Int Med J Exp Clin Res (2015) 21:1358–67. doi: 10.12659/MSM.893018 PMC4441288 25963880

[9] Li T Marinovich ML Houssami N. Digital breast tomosynthesis (3D mammography) for breast cancer screening and for assessment of screen-recalled findings: review of the evidence. Expert Rev Anticancer Ther (2018) 18(8):785–91. doi: 10.1080/14737140.2018.1483243 29847744

[10] Zackrisson S Lång K Rosso A Johnson K Dustler M Förnvik D. One-view breast tomosynthesis versus two-view mammography in the malmö breast tomosynthesis screening trial (MBTST): A prospective, population-based, diagnostic accuracy study. Lancet Oncol (2018) 19(11):1493–503. doi: 10.1016/S1470-2045(18)30521-7 30322817

[11] Zanardo M Cozzi A Trimboli RM Labaj O Monti CB Schiaffino S. Technique, protocols and adverse reactions for contrast-enhanced spectral mammography (CESM): A systematic review. Insights into Imaging (2019) 10(1):1–15. doi: 10.1186/s13244-019-0756-0 31376021 PMC6677840

[12] Dromain C Balleyguier C Muller S Mathieu MC Rochard F Opolon P. Evaluation of tumor angiogenesis of breast carcinoma using contrast-enhanced digital mammography. AJR Am J roentgenol (2006) 187(5):W528–37. doi: 10.2214/AJR.05.1944 17056886

[13] D’Orsi CJ Sickles EA Mendelson EB Morris EA. ACR BI-RADS® atlas, breast imaging reporting and data system. Reston, VA: American College of Radiology (2013).

[14] Lee CH Phillips CJ Sung JS Lewin JM Newell MS. Contrast enhanced mammography 2022, a supplement to ACR BI-RADS® atlas, breast imaging reporting and data system. Reston, VA: American College of Radiology (2013).

[15] NHSBSP Breast Screening Programme. Guidelines for non-operative diagnostic procedures and reporting in breast cancer screening. Sheffield: NHSBSP Publication No 50 (2001).

[16] Ferlay J Colombet M Soerjomataram I Parkin DM Piñeros M Znaor A. Cancer statistics for the year 2020: An overview. Int J Cancer (2021) 149(4):778–89. doi: 10.1002/ijc.33588 33818764

[17] Kornecki A. Current status of contrast enhanced mammography: A comprehensive review. Can Assoc Radiologists J = J l'Association Can Des radiologistes (2022) 73(1):141–56. doi: 10.1177/08465371211029047 34492211

[18] James JR Pavlicek W Hanson JA Boltz TF Patel BK. Breast radiation dose with CESM compared with 2D FFDM and 3D tomosynthesis mammography. AJR Am J roentgenol (2017) 208(2):362–72. doi: 10.2214/AJR.16.16743 28112559

[19] Jochelson MS Lobbes M. Contrast-enhanced mammography: State of the art. Radiology (2021) 299(1):36–48. doi: 10.1148/radiol.2021201948 33650905 PMC7997616

[20] Peppard HR Nicholson BE Rochman CM Merchant JK Mayo RC 3rd Harvey JA. Digital breast tomosynthesis in the diagnostic setting: Indications and clinical applications. Radiographics (2015) 35(4):975–90. doi: 10.1148/rg.2015140204 26024062

[21] Cozzi A Schiaffino S Fanizza M Magni V Menicagli L Monaco C. Contrast-enhanced mammography for the assessment of screening recalls: a two-centre study. Eur Radiol (2022), 1–12. doi: 10.1007/s00330-022-08868-3 PMC9668944 35648209

[22] Girometti R Linda A Conte P Lorenzon M De Serio I Jerman K. Multireader comparison of contrast-enhanced mammography versus the combination of digital mammography and digital breast tomosynthesis in the preoperative assessment of breast cancer. La Radiologia Med (2021) 126(11):1407–14. doi: 10.1007/s11547-021-01400-5 34302599

[23] Zuley ML Bandos AI Abrams GS Ganott MA Gizienski TA Hakim C. Contrast enhanced digital mammography (CEDM) helps to safely reduce benign breast biopsies for low to moderately suspicious soft tissue lesions. Acad Radiol (2020) 27(7):969–76. doi: 10.1016/j.acra.2019.07.020 31495761

[24] van der Molen AJ Reimer P Dekkers IA Bongartz G Bellin MF Bertolotto M. Post-contrast acute kidney injury–part 1: Definition, clinical features, incidence, role of contrast medium and risk factors. Eur Radiol (2018) 28(7):2845–55. doi: 10.1007/s00330-017-5246-5 PMC5986826 29426991

